# Plasmalogen Loss in Sepsis and SARS-CoV-2 Infection

**DOI:** 10.3389/fcell.2022.912880

**Published:** 2022-06-06

**Authors:** Daniel P. Pike, Reagan M. McGuffee, Elizabeth Geerling, Carolyn J. Albert, Daniel F. Hoft, Michael G. S. Shashaty, Nuala J. Meyer, Amelia K. Pinto, David A. Ford

**Affiliations:** ^1^ Edward A. Doisy Department of Biochemistry and Molecular Biology, Saint Louis University School of Medicine, St. Louis, MO, United States; ^2^ Center for Cardiovascular Research, Saint Louis University School of Medicine, St. Louis, MO, United States; ^3^ Department of Molecular Microbiology and Immunology, Saint Louis University School of Medicine, St. Louis, MO, United States; ^4^ Department of Internal Medicine, Division of Infectious Diseases, Allergy and Immunology, Saint Louis University School of Medicine, St. Louis, MO, United States; ^5^ Pulmonary, Allergy, and Critical Care Division, University of Pennsylvania Perelman School of Medicine, Philadelphia, PA, United States; ^6^ Center for Translational Lung Biology, University of Pennsylvania Perelman School of Medicine, Philadelphia, PA, United States

**Keywords:** sepsis, SARS-CoV-2, plasmalogen, infection, inflammation, lipidomics

## Abstract

Plasmalogens are plasma-borne antioxidant phospholipid species that provide protection as cellular lipid components during cellular oxidative stress. In this study we investigated plasma plasmalogen levels in human sepsis as well as in rodent models of infection. In humans, levels of multiple plasmenylethanolamine molecular species were decreased in septic patient plasma compared to control subject plasma as well as an age-aligned control subject cohort. Additionally, lysoplasmenylcholine levels were significantly decreased in septic patients compared to the control cohorts. In contrast, plasma diacyl phosphatidylethanolamine and phosphatidylcholine levels were elevated in septic patients. Lipid changes were also determined in rats subjected to cecal slurry sepsis. Plasma plasmenylcholine, plasmenylethanolamine, and lysoplasmenylcholine levels were decreased while diacyl phosphatidylethanolamine levels were increased in septic rats compared to control treated rats. Kidney levels of lysoplasmenylcholine as well as plasmenylethanolamine molecular species were decreased in septic rats. Interestingly, liver plasmenylcholine and plasmenylethanolamine levels were increased in septic rats. Since COVID-19 is associated with sepsis-like acute respiratory distress syndrome and oxidative stress, plasmalogen levels were also determined in a mouse model of COVID-19 (intranasal inoculation of K18 mice with SARS-CoV-2). 3 days following infection, lung infection was confirmed as well as cytokine expression in the lung. Multiple molecular species of lung plasmenylcholine and plasmenylethanolamine were decreased in infected mice. In contrast, the predominant lung phospholipid, dipalmitoyl phosphatidylcholine, was not decreased following SARS-CoV-2 infection. Additionally total plasmenylcholine levels were decreased in the plasma of SARS-CoV-2 infected mice. Collectively, these data demonstrate the loss of plasmalogens during both sepsis and SARS-CoV-2 infection. This study also indicates plasma plasmalogens should be considered in future studies as biomarkers of infection and as prognostic indicators for sepsis and COVID-19 outcomes.

## Introduction

Sepsis has been a major threat to global health over the past several decades. In the United States, approximately one million individuals are diagnosed with sepsis annually, with mortality estimated between 12 and 25 percent (Mayr et al., 2014; Paoli et al., 2018). An estimated 20 percent of all deaths globally were attributed to sepsis (Rudd et al., 2020). The more severe septic shock has an estimated 38 percent mortality, and half of all Americans who die in the hospital are diagnosed with sepsis (Liu et al., 2014; Vincent et al., 2019). Sepsis occurs when an infection triggers a dysregulated host immune response, leading to systemic microcirculatory and immune dysfunction. This dysregulated inflammatory response in the microvasculature leads to direct damage of cells from reactive oxygen species and other inflammatory mediators, activation of the coagulation cascade, vasodilation, and tissue hypoxia with subsequent mitochondrial dysfunction. This complex system culminates in life-threatening organ injury and metabolic derangements (Chuang et al., 2006; Robertson and Coopersmith, 2006; Galley, 2011; Angus and van der Poll, 2013; Delano and Ward, 2016; Singer et al., 2016; Prauchner, 2017). Lipids and lipid-related signaling pathways have been investigated as mediators, potentially at the blood-endothelial interface during sepsis (Amunugama et al., 2021a). Specific lipids may also have prognostic value as biomarkers in sepsis (Meyer et al., 2017; Mecatti et al., 2018; Mecatti et al., 2020; Wang et al., 2020; Amunugama et al., 2021a). Additionally, a major cause of COVID-19 mortality is sepsis-associated acute respiratory distress syndrome (ARDS). Similar to sepsis, lipids have been investigated as important mediators and biomarkers in COVID-19 (Tanner et al., 2014; Aktepe et al., 2015; Villareal et al., 2015; Jean Beltran et al., 2018; Fernández-Oliva et al., 2019; Sviridov et al., 2020; Casari et al., 2021; Mesquita et al., 2021; Theken et al., 2021).

Plasmalogens comprise a significant fraction of the lipid content in the plasma, immune cells, and endothelium (Chilton and Murphy, 1986; Chilton and Connell, 1988; Kayganich and Murphy, 1992; Murphy et al., 1992; Bräutigam et al., 1996). There is considerable diversity in plasmalogen molecular species. In general, plasmalogens contain either phosphocholine or phosphoethanolamine at the *sn*-3 position of the glycerol backbone. The vinyl ether aliphatic group attached to the glycerol backbone predominantly contains sixteen and eighteen carbon groups. Recently we have also shown neutrophil plasmalogens contain vinyl ether groups that are greater than twenty carbons in length (Amunugama et al., 2021b). Plasmalogens have been suggested to have important roles in biological membranes, which are due, in part, to their unique packing in membranes compared to diacyl phospholipids (Han and Gross, 1990; Han and Gross, 1991). Plasmalogens have been shown to have roles in synaptic fusion, cholesterol efflux, lipid rafts, and transmembrane protein function (Glaser and Gross, 1994; Ford and Hale, 1996; Mandel et al., 1998; Pike et al., 2002). Plasmalogens likely have key roles in inflammation at several levels. Plasmalogens are plasma-borne antioxidants and have been shown to protect endothelium from oxidative stress (Vance, 1990; Zoeller et al., 1999). The vinyl ether bond of plasmalogens is susceptible to attack by reactive species, and this propensity suggests that these lipids can protect cells by scavenging reactive oxygen species (Zoeller et al., 1988; Reiss et al., 1997; Zoeller et al., 1999; Zoeller et al., 2002; Dean and Lodhi, 2018). Additionally, plasmalogens have been shown to have a key role in macrophage phagocytosis (Rubio et al., 2018). Furthermore, plasmalogens are enriched with arachidonic acid and docosahexaenoic acid at the *sn*-2 position, and their metabolism by phospholipases leads to the mobilization of these fatty acids and their subsequent oxidation to bioactive eicosanoids and resolvins (Paul et al., 2019). Collectively, the roles of plasmalogens in membrane molecular dynamics, as antioxidants, and as precursors of bioactive lipids indicate they may be important in inflammation associated with disease and infection.

Plasma plasmalogen levels have been shown to decrease during inflammation such as during endotoxemia (Ifuku et al., 2012), Parkinson’s disease (Dragonas et al., 2009; Fabelo et al., 2011), and lupus (Hu et al., 2016). Several of these previous studies (Dragonas et al., 2009; Hu et al., 2016) have suggested the loss of plasmalogens during Parkinson’s disease and lupus is due to the associated oxidative stress. Surprisingly only one study has investigated plasmalogen loss during human sepsis, which also attributed plasmalogen loss to oxidative stress (Brosche et al., 2013). This study was limited to measuring dimethyl acetals as a measure of plasmalogen levels and was performed in a limited number of geriatric septic patients. In addition to sepsis, several investigations have emerged over the past 2 years demonstrating plasma plasmalogen levels in humans with severe COVID-19 are decreased (Schwarz et al., 2021; Snider et al., 2021). The loss of plasmalogens and other phospholipids enriched with arachidonic acid and docosahexaenoic acid as well as increased secretory phospholipase A_2_ (Snider et al., 2021) in COVID-19 patients support an important role for plasmalogens as precursors of oxylipids.

We have previously shown the plasmalogen vinyl ether bond is targeted by neutrophil-derived HOCl (a product of myeloperoxidase activity) resulting in 2-chlorofatty aldehyde and 2-chlorofatty acid production (Albert et al., 2001; Thukkani et al., 2002; Anbukumar et al., 2010). Furthermore, increased 2-chlorofatty acid plasma levels associate with ARDS-caused mortality in human sepsis (Meyer et al., 2017). 2-Chlorofatty acids are also elevated in the plasma and several organs in rats subjected to cecal slurry sepsis (Pike et al., 2020). Since plasmalogens are the precursors of chlorinated lipid production during sepsis and since limited molecular detail is known about human plasma plasmalogen loss during sepsis, in the present study we have investigated plasma plasmalogen levels in human sepsis patients. Furthermore, we have employed the rat cecal slurry sepsis model to identify both plasma plasmalogen loss as well as changes in liver and kidney plasmalogen levels during sepsis. Lastly, we examined changes in plasmalogen levels in plasma and lung in mice challenged with SARS-CoV-2. Collectively, these studies show the loss of plasmalogens during sepsis and SARS-CoV-2 infection with new detail into changes in plasma molecular species, as well as changes in organs in rodent models of sepsis and COVID-19.

## Materials and Methods

### Human Plasma Specimens and Analysis

Sepsis plasma samples were obtained from subjects admitted to the intensive care unit (ICU) with suspected infection and acute organ dysfunction (sepsis) at day 7 in the ICU. The cohort has been previously described (Reilly et al., 2018). The cohort study is approved by the University of Pennsylvania institutional review board (IRB protocol #808542), and all subjects or their proxies provided informed consent to participate. Control healthy plasma samples were obtained at Saint Louis University under IRB protocol 26646. Plasma samples were stored in aliquots to minimize freeze thaw cycles to two times or less.

### Rat Cecal Slurry Studies

Rats were supplied from Envigo (Harlan—Indianapolis, IN, United States). All rats were young adult male Sprague-Dawley weighing between 270–330 g (8–12 weeks old). All animals were maintained in a temperature and humidity-controlled room with a 12 h light/dark cycle and unrestricted access to chow and water. Upon arrival to Saint Louis University, rats were acclimated to the environment for at least a week prior to experiments. All animal experiments were conducted with the approval of the Institutional Animal Care and Use Committee at Saint Louis University. Cecal slurry (CS) was prepared from cecal contents of donor male Sprague-Dawley rats as previously detailed (Pike et al., 2020). Prior to ip CS administration for sepsis studies, aliquots of CS were thawed quickly in warm water. Rats were administered 15 ml/kg CS or 15% glycerol vehicle control (ip) in a total volume of 20 ml/kg, with the remaining 5 ml/kg being sterile saline (B Braun Medical, Bethlehem, PA, United States). At the time of CS administration, animals were administered a concurrent 30 ml/kg dose of subcutaneous sterile saline. Eight hours following CS treatment, 25 mg/kg ceftriaxone (Hospira) in sterile saline was administered intramuscularly in the hind limb in a 1 ml/kg volume. A second subcutaneous 30 ml/kg dose of sterile saline was administered concurrently with the ceftriaxone in order to simulate treatment of human sepsis with crystalloid and antibiotics. 20 h following CS injection, rats were euthanized, and organs were collected, which were immediately frozen on dry ice. Blood was collected *via* cardiac puncture, and plasma was immediately prepared and then stored at −80°C. Plasma preparation and storage was achieved within 30–45 min of the blood draw. Plasma samples were stored in aliquots to minimize freeze thaw cycles to two times or less. Rats were euthanized by injecting 0.5 ml Somnasol (390 mg/ml sodium pentobarbital and 50 mg/ml phenytoin sodium), ip followed by thoracotomy.

### Mouse SARS-CoV-2 Infection Studies

K18 mice (JAX strain 034860, human angiotensin converting enzyme 2 (hACE2 transgenic)) were supplied from the Jackson Laboratory (Bar Harbor, MA, United States). All mice were young adult females weighing between 25–30 g (∼9 weeks old). All animals were maintained in a temperature and humidity-controlled room with a 12 h light/dark cycle and unrestricted access to chow and water. Upon arrival to Saint Louis University, mice were acclimated to the ABSL-3 environment for at least a week prior to experiments. All animal experiments were conducted with the approval of the Institutional Animal Care and Use Committee at Saint Louis University. K18 mice were either mock infected or infected with 1 × 10^4^ focus forming units (FFU) of the beta variant B.1.351 of SARS-CoV-2 intranasally (20 μl). The beta variant B.1.351 of SARS-CoV-2 was obtained from BEI Resources (#NR55282). Tissues and plasma were collected from euthanized mice three- or 4-days following infection. Tissue homogenates were prepared for analyses of either viral burden, cytokine mRNA, or lipids. SARS-CoV-2 viral burden was measured by focus forming assays (FFAs) using Vero E6 cells transfected with hACE2 and TMPRSS2 as we have previously described (Geerling et al., 2022). Inflammatory cytokine levels were measured *via* qRT-PCR using Taqman primer and probe sets from IDT as previously described (Geerling et al., 2021).

### Lipid Analysis

Tissue and plasma lipids were extracted in the presence of internal standards (see Supplementary Table S1) by a modified Bligh-Dyer extraction as previously described (Bligh and Dyer, 1959; Maner-Smith et al., 2020; Pike et al., 2020; Amunugama et al., 2021b). Individual choline and ethanolamine glycerophosphospholipids were detected using selected reaction monitoring (see Supplementary Table S1 for transitions) with an Altis TSQ mass spectrometer equipped with a Vanquish UHPLC System (Thermo Scientific) with isotopomer corrections for each target molecular species compared to the respective internal standard. Lipids were separated on an Accucore^TM^ C30 column 2.1 mm × 150 mm (Thermo Scientific) with mobile phase A comprised of 60% acetonitrile, 40% water, 10 mM ammonium formate, and 0.1% formic acid and mobile phase B comprised of 90% isopropanol, 10% acetonitrile with 2 mM ammonium formate, and 0.02% formic acid. Initial conditions were 30% B with a discontinuous gradient to 100% B at a flow rate of 0.260 ml/min. Plasmalogen molecular species were identified by acid lability and fatty acid aliphatic group identification under identical conditions employed using the TSQ mass spectrometer but using a Q-Exactive mass spectrometer with choline glycerophospholipids detected in negative ion mode.

### Statistics

Student’s t-test was used to compare two groups in rat CS and K18 mouse SARS-CoV-2 infection studies. Plasma concentrations were compared between healthy control subjects and sepsis subjects by Wilcoxon rank sum test.

## Results

### Alterations in Plasma Plasmalogen and Diacyl Phospholipids in Human Sepsis

**FIGURE 1:**
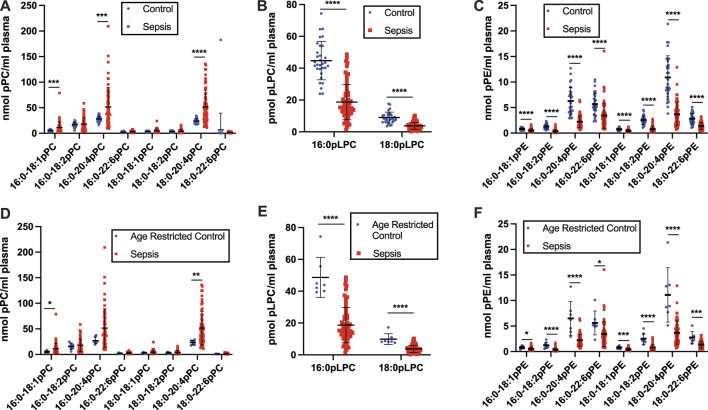
Loss of plasma plasmenylethanolamine (pPE) and lysoplasmenylcholine (pLPC) in human sepsis. Plasma was collected from 31 healthy humans (control) and 63 ICU patients with sepsis following 7 days in the ICU. Lipids were extracted and plasmalogen levels were quantitated as described in “Materials and Methods.” Plasma plasmenylcholine (pPC) **(A,D)**, pLPC **(B,E)**, and pPE **(C,F)** are compared between the control and sepsis cohorts **(A–C)** and an age restricted control and sepsis cohorts **(D–F)**. *, **, ***, and **** indicate *p* < 0.05, 0.01, 0.001, and 0.0001, respectively, for comparisons between cohorts. Mean and standard deviation values are indicated for each molecular species and condition.

**FIGURE 2:**
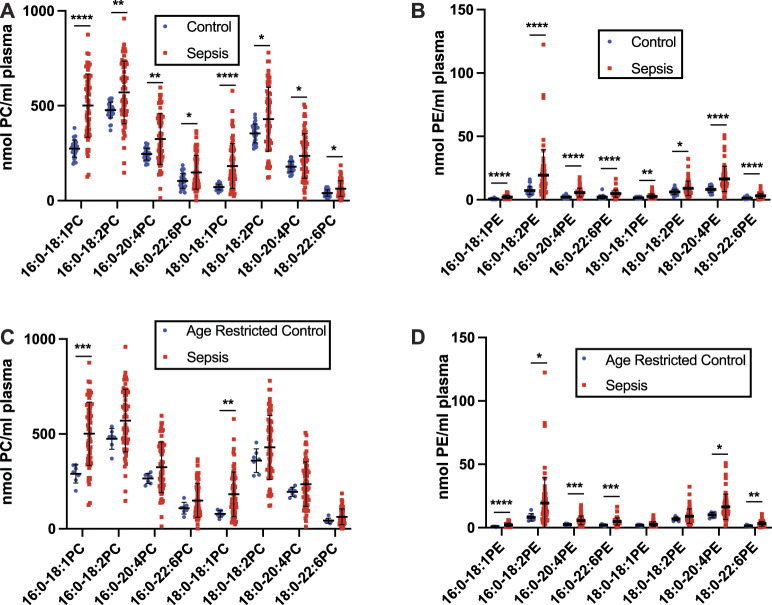
Increases in plasma phosphatidylcholine (PC) and phosphatidylethanolamine (PE) in human sepsis. Plasma was collected from 31 healthy humans (control) and 63 ICU patients with sepsis following 7 days in the ICU. Lipids were extracted and plasmalogen levels were quantitated as described in “Materials and Methods.” Plasma PC **(A,C)** and PE **(B,D)** are compared between the control and sepsis cohorts **(A,B)** and an age restricted control and sepsis cohorts **(C,D)**. *, **, ***, and **** indicate *p* < 0.05, 0.01, 0.001, and 0.0001, respectively, for comparisons between cohorts. Mean and standard deviation values are indicated for each molecular species and condition.

Human geriatric septic patients have previously been shown to have decreased plasma plasmalogen levels as determined by assessing dimethyl acetals of plasmalogens by gas chromatography. These analyses did not identify the lipid class (choline or ethanolamine) of the plasmalogen pool or the molecular species that decrease during sepsis. Additionally, we have previously shown plasma 2-chloropalmitic acid levels are increased in human sepsis and associate with ARDS-caused mortality (Meyer et al., 2017). 2-Chloropalmitic acid is derived from 2-chloropalmitaldehyde produced by the action of HOCl targeting the vinyl ether bond of plasmalogens (Albert et al., 2001; Thukkani et al., 2002; Anbukumar et al., 2010). Accordingly, we performed a detailed study of plasma plasmalogens in septic humans. The plasma specimens of patients in this study are from septic patients collected following 7 days in the ICU. The average age of these patients is 59.8 years. Interestingly, data shown in Figure 1A show levels of plasma plasmenylcholine (pPC) molecular species either were unchanged or increased in septic patients compared to control subjects. Since the control cohort age was younger than the sepsis group (Table 1), we also compared changes in plasma pPC levels between the sepsis cohort and an age restricted subgroup of the control subjects to test a cohort that was more closely aligned in age with the sepsis cohort (Figure 1D). A similar pattern of either increased or unchanged levels of pPC was observed in the septic patients compared to the age restricted controls to sepsis. The two pPC molecular species elevated in sepsis were 16:0-18:1 pPC and 18:0-20:4 pPC (x:y-x:y where x# of carbons and y# of double bonds in aliphatic groups at the *sn*-1 and *sn*-2 position, respectively). In contrast, significant decreases were observed with plasma 16:0 and 18:0 lysoplasmenylcholine (pLPC) in septic subjects with comparisons to both the unrestricted control group **(** Figure 1B), as well as the age restricted control group (Figure 1E). Furthermore, all plasma plasmenylethanolamine (pPE) molecular species in our targeted analyses were significantly decreased in the septic patient cohort in comparison to both the unrestricted control group (Figure 1C), as well as the age restricted control group (Figure 1F). In contrast to pPE, plasma levels of diacyl phosphatidylethanolamine (PE), as well as phosphatidylcholine (PC), were increased in the sepsis cohort in comparisons to both the unrestricted and age restricted cohorts **(** Figures 2A–D).

**TABLE 1:** Clinical characteristics of the sepsis and control population.

	Sepsis (*n* = 63)	Controls (*n* = 31)	Age restricted controls (*n* = 7)
Age, years	59.8 ± 12.7	38.2 ± 15.1	56.6 ± 8.4
Female sex (N, %)	25, 39.6%	Not available	Not available
APACHE III score^a^	85 (68, 107)	—	—
Diabetes (N, %)	19, 30.2%		
Solid organ malignancy (N, %)	12, 19%		
Hematologic malignancy (N, %)	20, 31.7%		
Mortality at 30 days (N, %)	17, 27%		

^a^ The acute physiology and chronic health examination (APACHE) III score is displayed as median (interquartile range) due to a skewed distribution.

### Alterations in Plasmalogen and Diacyl Phospholipids in Rodent Sepsis

**FIGURE 3:**
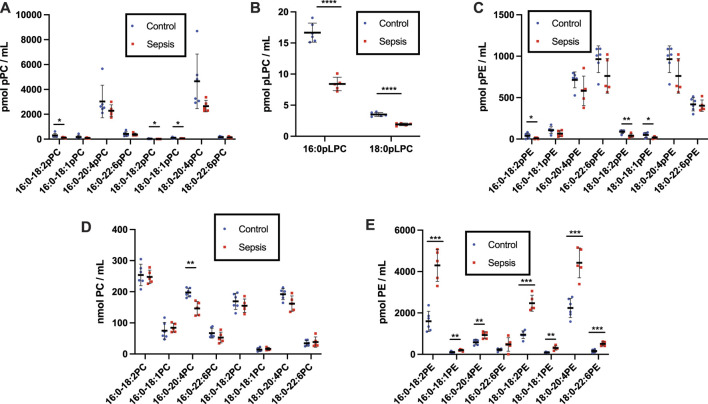
Loss of plasma plasmalogens and increases in diacyl PC and PE in septic rats. Rats were injected with cecal slurry (sepsis) (*n* = 5) or vehicle (control) (*n* = 6) and were subsequently treated with fluid replacement and ceftriaxone 8 h following cecal slurry injection as described in “Materials and Methods.” Plasma was collected 20 h following cecal slurry or vehicle treatment, and lipids were extracted. Plasmalogen levels were quantitated as described in “Materials and Methods.” Plasma pPC, pLPC, pPE, PC, and PE are shown in **(A–E)**, respectively. *, **, ***, and **** indicate *p* < 0.05, 0.01, 0.001, and 0.0001, respectively, for comparisons between control and septic rats. Mean and standard deviation values are indicated for each molecular species and condition.

To gain further insights into alterations in plasmalogens, as well as diacyl phospholipids, during sepsis we examined both plasma and tissue changes in these phospholipids in the cecal slurry (CS) rodent model of sepsis. Previous studies have demonstrated under the CS infection conditions followed by antibiotic treatment 8 h post infection employed in these studies, rats survive at least 20 h and have increased plasma 2-chlorofatty acid levels in comparison to vehicle treated rats (Pike et al., 2020). pPC was identified as the most abundant plasmalogen class in both control and sepsis rat plasma compared to pPE. (Figures 3A,C). Plasma plasmalogen loss was observed in CS treated rats compared to vehicle injected rats. Plasma 16:0-18:2, 18:0-18:2, and 18:0-18:1 pPC levels were decreased in septic rats 20 h post infection (Figure 3A **)**. Similar to human sepsis, both 16:0 and 18:0 pLPC levels were decreased in septic rats in comparison to control vehicle-treated rats **(** Figure 3B). In contrast to human sepsis, the predominant species of plasma pPE levels were not significantly decreased in rat sepsis, however less abundant species such as 16:0-18:2, 18:0-18:2, and 18:0-18:1 pPE did significantly decrease (Figure 3C). For the diacyl species, sepsis resulted in a decrease of only 16:0-20:4 PC in rat plasma (Figure 3D **)**. In stark contrast to the drop in plasma pPE levels, all diacyl PE levels were significantly increased (Figure 3E **)**.

**FIGURE 4:**
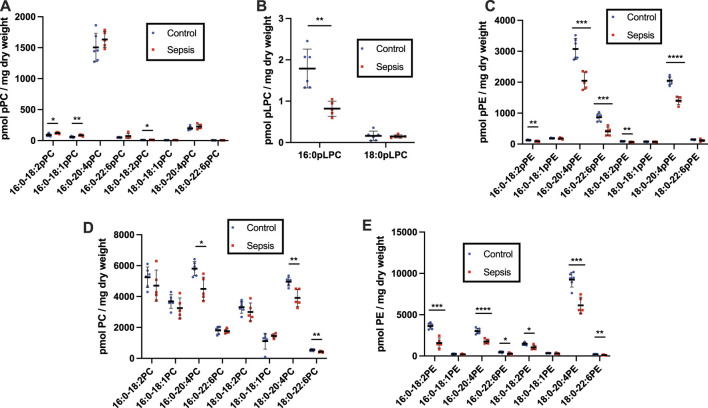
Alterations in kidney diacyl and plasmalogen phospholipids during rat sepsis. Rats were injected with cecal slurry (sepsis) (*n* = 5) or vehicle (control) (*n* = 6) as described in Figure 3. Kidneys were collected 20 h following cecal slurry or vehicle treatment, and lipids were extracted. Plasmalogen levels were quantitated as described in “Materials and Methods.” Kidney pPC, pLPC, pPE, PC, and PE are shown in **(A–E)**, respectively. *, **, ***, and **** indicate *p* < 0.05, 0.01, 0.001, and 0.0001, respectively, for comparisons between control and septic rats. Mean and standard deviation values are indicated for each molecular species and condition.

**FIGURE 5:**
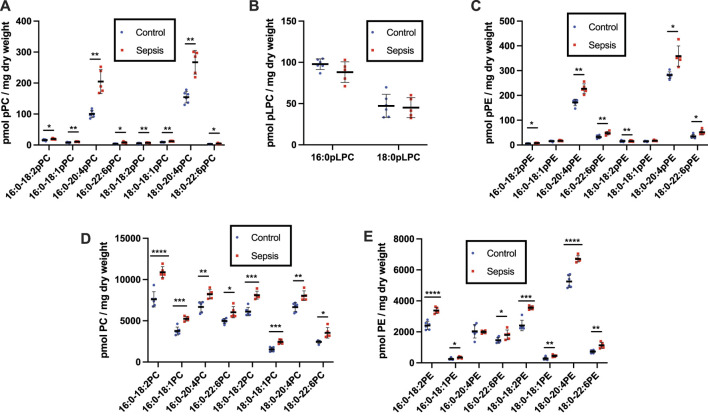
Alterations in liver diacyl and plasmalogen phospholipids during rat sepsis. Rats were injected with cecal slurry (sepsis) (*n* = 5) or vehicle (control) (*n* = 6) as described in Figure 3. Liver was collected 20 h following cecal slurry or vehicle treatment, and lipids were extracted. Plasmalogen levels were quantitated as described in “Materials and Methods.” Liver pPC, pLPC, pPE, PC, and PE are shown in **(A–E)**, respectively. *, **, ***, and **** indicate *p* < 0.05, 0.01, 0.001, and 0.0001, respectively, for comparisons between control and septic rats. Mean and standard deviation values are indicated for each molecular species and condition.

Previously in this rodent model we identified the kidney and liver as primary sites of organ failure based on loss of permeability barrier function as assessed by Evans blue extravasation (Pike et al., 2020). Additionally, both liver and kidney levels of 2-chlorofatty acids were previously shown to be increased in this sepsis model (Pike et al., 2020). 2-Chlorofatty acids are produced as a result of neutrophil-derived HOCl targeting plasmalogens (Thukkani et al., 2002; Anbukumar et al., 2010). Accordingly, we examined plasmalogen levels in the kidney and liver of CS infected rats. In contrast to plasma, pPE is the predominant plasmalogen class in both rat kidney and liver compared to pPC (Figures 4, 5). Multiple pPE molecular species in the rat kidney were significantly decreased in septic rats, including the predominant 16:0-20:4 and 18:0-20:4 pPE species (Figure 4C). Renal 16:0 pLPC was also significantly decreased in sepsis (Figure 4B). Meanwhile, some less predominant renal pPC levels were increased (Figure 4A). In contrast to changes in rat plasma and kidney plasmalogens, as well as in human plasma, several liver plasmalogens increased during rat sepsis. All pPC species significantly increased, including the predominant 16:0-20:4 pPC and 18:0-20:4 pPC species, in livers of CS elicited septic rats (Figure 5A). 16:0-20:4 pPE and 18:0-20:4 pPE, among others, also were significantly increased in livers from septic rats compared to control rats (Figure 5C). Further in contrast to changes in the plasma and kidney, there was no significant difference in pLPC levels in livers from septic rats compared to those of control rats (Figure 5B). Diacyl species were measured in the kidney and liver as well. In the kidney, multiple species of diacyl PC and PE were significantly decreased (Figures 4D,E). While in the liver, CS-elicited sepsis resulted in increases in both diacyl PC and PE (Figures 5D,E).

### Plasmalogens in SARS-CoV-2 Infected K18 Mice

**FIGURE 6:**
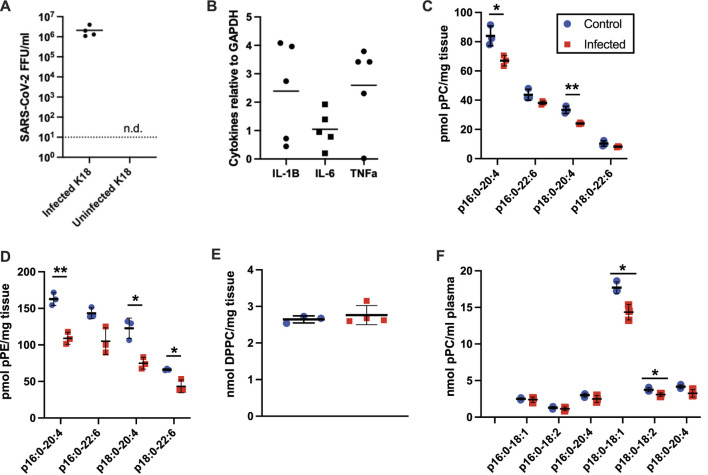
Plasmalogen changes in K18 mice infected with the beta B.1.351 SARS-CoV-2 variant. K18 mice were either mock infected (blue circles) or infected (red squares) with 10^4^ FFU of SARS-CoV-2, 20 ul, IN. 3 days post infection, lungs **(C–E)** and plasma **(F)** were collected for lipidomics analyses (*n* = 3 for both conditions). *, **, and *** indicate *p* < 0.05, 0.01, and 0.001, respectively, for comparisons between mock and virus infected mice. **(A)** SARS-CoV-2 titers in lungs of infected (*n* = 4) and uninfected (*n* = 4) mice from this study. N.d. indicates titers were not observed in the uninfected lungs. **(B)** Pro-inflammatory cytokine mRNA was detected in the infected lungs (*n* = 4 or 5) but were not detected in the uninfected lungs. Mean and standard deviation values are indicated for each molecular species and condition.

Since plasmalogens have been shown to decrease in the plasma of humans with severe COVID-19 (Schwarz et al., 2021; Snider et al., 2021) and SARS-CoV-2 infection leads to a form of sepsis-associated ARDS, we investigated the role of airway infection with SARS-CoV-2 in K18-hACE2 transgenic mice. The human keratin 18 promoter (K18) in K18 mice directs human ACE2 expression in the epithelium, which is important as SARS-CoV-2 infections tend to begin in airway epithelia. Three days following nasal inoculation with SARS-CoV-2 a robust viral burden was observed in the lung (Figure 6A), which is similar to findings by others (Zheng et al., 2021). The associated cytokine storm of SARS-CoV-2 infection was confirmed with increases in interleukin-1β (IL-1B), interleukin-6 (IL-6) and tumor necrosis factor-α (TNF-α) mRNA expression in lung tissue **(** Figure 6B). These cytokine mRNAs were not detected in mock-infected lung (data not shown). 16:0-20:4 pPC and 18:0-20:4 pPC levels in the lung were selectively decreased in SARS-CoV-2 infected K18 mice **(** Figure 6C). Additionally, both 16:0-20:4 pPE and 18:0-20:4 pPE, as well as 18:0-22:6 pPE, were decreased in the lung of SARS-CoV-2 mice **(** Figure 6D). As in rat tissues, pPE levels were higher than that of pPC in the mouse lung. We also assessed the major lung lipid, 1,2-dipalmitoyl-*sn*-glycero-3-phosphocholine (DPPC) in the lungs, which is the major phospholipid component of surfactant. Lung DPPC levels were not altered in SARS-CoV-2 infected mice **(** Figure 6E). Changes in plasma plasmalogen levels were only modestly decreased in SARS-CoV-2 infected mice (Figure 6F).

## Discussion

Plasmalogens are a lipid subclass characterized by a vinyl ether linked aliphatic group attached to the *sn*-1 position of glycerol, a fatty acid esterified at the *sn*-2 position and, in general, either phosphoethanolamine or phosphocholine at the *sn*-3 position. The *sn*-2 fatty acid of plasmalogens is enriched with arachidonic acid in many mammalian tissues and thus one role of plasmalogens has been described as a storage depot for arachidonic acid that is released during inflammation (Chilton and Connell, 1988; Ford and Gross, 1989; Braverman and Moser, 2012). The *sn*-1 vinyl ether is a target for reactive oxygen species leading to the release of free fatty aldehydes that subsequently can be metabolized to free fatty acids (Khaselev and Murphy, 1999; Stadelmann-Ingrand et al., 2001). The reaction of reactive oxygen species with the vinyl ether is a terminal event for ROS and thus is considered an antioxidant activity. Multiple studies have shown plasmalogens protect tissues and cells from reactive oxygen species and oxidative stress. Cells deficient in plasmalogens are susceptible to free radical-mediated toxicity (Morand et al., 1988; Zoeller et al., 1988). Furthermore, supplementing cells with precursors to plasmalogens has been shown to protect cells from reactive oxygen species including during hypoxic damage to endothelial cells (Zoeller et al., 1999). Collectively, the abundance of arachidonic acid esterified to plasmalogens that can be mobilized for eicosanoid production and the susceptibility of the vinyl ether to oxidative stress suggest plasmalogens may have important roles in infection and inflammation. Plasma plasmalogen depletion has also been demonstrated in humans with Parkinson’s disease, Alzheimer’s disease, lupus and endotoxemia (Dragonas et al., 2009; Fabelo et al., 2011; Ifuku et al., 2012; Hu et al., 2016; Su et al., 2019). In the present study we provide further support for the involvement of plasmalogens in inflammation by providing molecular detail to changes in plasmalogen levels both in plasma and in organs during sepsis as well as SARS-CoV-2 infection.

**FIGURE 7:**
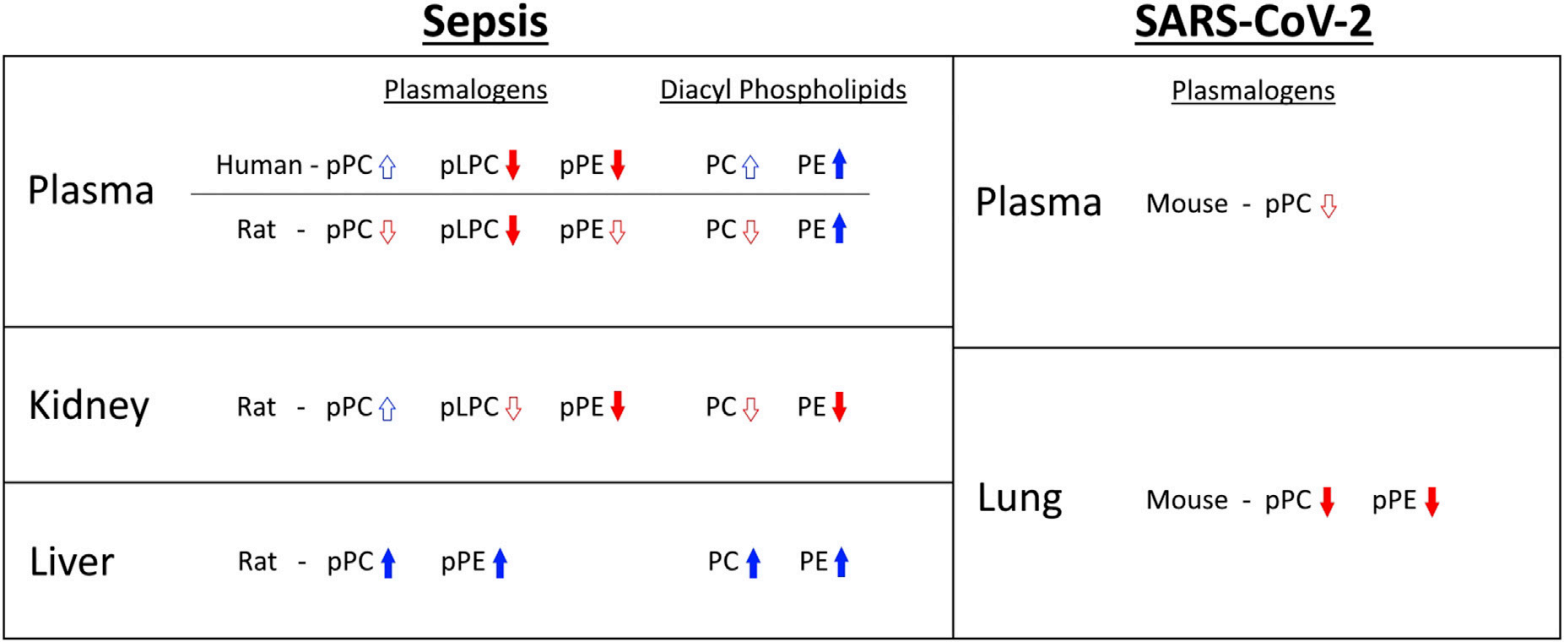
Summary of plasmalogen and diacyl phospholipid changes observed in sepsis and SARS-CoV-2 infection. Arrow outlines indicate that fewer than half of reported species show statistically significant increase or decrease. Solid arrows indicate that at least half of reported species show statistically significant increase or decrease. For human sepsis, only trends in age restricted data are shown.

Previous studies showed the 16:0 dimethyl acetal derivative of plasmalogens containing a sixteen-carbon vinyl ether aliphatic group bound to the glycerol backbone are decreased 55% in plasma of twenty geriatric septic patients compared to age-matched healthy subjects (Brosche et al., 2013). In this previous study, data for 18:0 dimethyl acetals were not reported and changes in 16:0 dimethyl acetal were from patient plasma collected within 24 h of severe sepsis diagnosis. Human plasma pPC levels are ∼8–10 fold greater than pPE levels, and pPC is highly enriched in molecular species containing a sixteen-carbon vinyl ether aliphatic group bound to the glycerol backbone, suggesting the plasma plasmalogens that decreased in geriatric sepsis patients (Brosche et al., 2013) are from pPC pools. In contrast to this previous study, our findings from the MESSI cohort were from patient plasma collected 7d following ICU admission for sepsis. This difference in time for plasma collection prevents direct comparisons to the previously reported study (Brosche et al., 2013). However, in the present studies pPE molecular species containing 16:0 vinyl ether groups, as well as 16:0 pLPC, were decreased in the human sepsis cohort. Plasma pPE species containing 18:0 vinyl ether groups were also significantly decreased in septic subjects investigated in our study. Future studies should be directed at determining details of plasmalogen loss at 24 h and examine longitudinal changes in plasmalogen loss. It will also be interesting to compare changes in human plasmalogen molecular species at 24 h to the changes we observed in the rat plasma plasmalogen molecular species that changed 20 h post CS injection. Interestingly with rat sepsis, plasma plasmalogen loss at 20 h decreased in several pPC and pPE species as well as pLPC. A summary of levels of plasmalogen and diacyl species shows a general downward trend in plasmalogen levels in sepsis, excluding livers from of septic rats **(** Figure 7 **)**. In particular, this summary highlights the many differences in changes elicited during sepsis between plasmalogen and diacyl phospholipid levels depending on the tissue and particular phospholipid class. One of the more striking observations is the loss of pPE in plasma in contrast to increases in diacyl PE during sepsis in both humans and rats.

The mechanisms responsible for plasma pLPC and pPE loss during sepsis are not known, but several mechanisms seem likely. One mechanism is that loss of plasmalogen is due to oxidative stress during sepsis. We have previously shown plasma 2-chlorofatty acid levels are elevated in human sepsis (Meyer et al., 2017; Amunugama et al., 2021b). Furthermore, in this rat sepsis model there are increased levels of 2-chlorofatty acid levels (Pike et al., 2020), which is derived from plasmalogens (Albert et al., 2001; Thukkani et al., 2002; Amunugama et al., 2021b). During sepsis the tissue plasmalogen pool or the specific plasmalogen molecular species targeted by HOCl has not been determined. In this respect it could be speculated that the impressive loss of plasma pLPC, which is overall a small pool of the total plasmalogen, could be responsible for the nanomolar levels of 2-chlorofatty acid observed during sepsis. It is also possible that the loss of plasmalogens is due to the activation of phospholipases. It has been suggested that phospholipase A_2_-mediated release of arachidonic acid from plasmalogens is important in the production of oxylipids in COVID-19 (Schwarz et al., 2021; Snider et al., 2021). The phospholipase A_2_ mechanisms may be directly responsible for pPE loss. It is also possible pLPC loss is due to either accelerated use as an acceptor by acyltransferases leading to conserved levels of pPC despite putative oxidative loss or tissue uptake during sepsis. Another possibility is pPE and pLPC decrease as a result of reduced release from the liver and vascular endothelium. In human sepsis, HDL-cholesterol decreases (Vavrova et al., 2016; Tanaka et al., 2019), which may also be due to decreased secretion from the liver. Decreased plasma plasmalogens and increased liver plasmalogens during sepsis are similar to plasmalogen changes in *H-Lrpprc* mice, a mouse model of the monogenic form of the mitochondrial disease, Leigh syndrome (Ruiz et al., 2019). In *H-Lrpprc* mice, hepatic *Far1* and *Agps* are also elevated suggesting decreased plasma plasmalogen levels mediate a feedback system to increase liver plasmalogen biosynthesis. Such a feedback system may also be responsible for elevated liver plasmalogen levels in livers during sepsis. It will be interesting in future studies to examine *Agps* and *Far1* as well as differences in the levels of the plasmalogen precursors, alkyl ether lipids, in the livers from septic and control rats.

The possibility that pLPC is a circulating precursor to enrich plasmalogens in endothelium is intriguing. Plasmalogen enhancement in isolated cell studies protects cells from oxidative stress (Zoeller et al., 1999). Additionally, several studies have investigated plasmalogen precursors as a potential treatment in inflammatory diseases (Bozelli and Epand, 2021; Paul et al., 2021). Enhancing plasmalogen levels is difficult since dietary consumption of plasmalogens is reduced due to the acidic environment of the gastrointestinal tract. Using acid-stable precursors such as alkyl ether lipids will raise plasmalogen levels over time following desaturation of the alkyl ether bond to the vinyl ether. However, under acute conditions such as sepsis, the conversion of an alkyl ether to plasmalogens likely will be very slow. On the other hand, circulating pLPC already has the vinyl ether bond and lysolipids are rapidly incorporated into cells. It will be important in the future to determine the source of circulating pLPC under physiological conditions as well as during sepsis. It could be envisaged that pLPC is a product of lipoprotein-associated pPC hydrolysis by either secretory phospholipase A_2_ or lipoprotein lipase. During sepsis pLPC levels potentially are dependent on a combination of oxidation of pPC or pLPC and pPC hydrolysis. Finally, the role of pLPC during sepsis needs to be further considered as a biomarker of outcomes. Similarly, the role of other plasmalogens, as well as the relationship of plasma plasmalogen levels with changes in plasma 2-chlorofatty acid levels, need to be considered as outcome predictors. The relationship of plasmalogen and chlorinated lipid levels may also allow distinction of changes in these lipids with greater specificity to infection compared to other disease states associated with only decreased plasma plasmalogen levels with the exception of lupus (Dragonas et al., 2009; Fabelo et al., 2011; Ifuku et al., 2012; Mahieu et al., 2014; Hu et al., 2016; Paul et al., 2019; Su et al., 2019).

The studies herein show plasmalogen loss during sepsis. However, there are several limitations to these studies. In the human studies we analyzed differences between septic humans and healthy control humans. Our healthy cohort average age was thirty-eight while the sepsis group was sixty. To overcome this limitation, we selected the oldest individuals (*n* = 7) in the healthy group and assessed differences in this control subset compared to the larger group of septic subjects (Figures 1D–F, 2; Table 1). These additional analyses indicated plasma pLPC and pPE levels were reduced in the sepsis cohort when compared to this age-aligned control subgroup. Another limitation is that we have no data on the sex of individuals in our healthy cohort, while our sepsis cohort was comprised of 40% females. Our rat studies focused on changes occurring only in male rats and 20 h following cecal slurry injection. Thus, comparisons of rat specimens to human specimens were collected at different times and sex differences were not a parameter in the rat studies. It should also be appreciated that plasma levels of plasmalogens were considerably different in healthy controls due to the inherent differences in plasmalogen levels in man versus rat. Nevertheless, both human and rat sepsis led to decreases in plasma plasmalogen levels, and the rat studies afforded the opportunity to investigate changes in plasmalogen levels in the liver and kidney during sepsis. There were also limitations to the SARS-CoV-2 infection studies when comparisons are made to the rat and human sepsis studies. The SARS-CoV-2 infection studies were a viral infection elicited by airway inoculation to transgenic mice expressing the hACE2 receptor in all epithelial cells. Humans do not express ACE2 in all epithelial cells. Furthermore, these studies were performed only in female mice due to availability of genotyped mice for this study. Future studies are needed to consider sex as a parameter in both SARS-CoV-2 infected mice and rat cecal slurry sepsis. Compared to the unknown time for human sepsis beginning and the known time for CS injection, mouse infections with SARS-CoV-2 leading to pulmonary inflammation require time for viral replication to elicit injury which is typically 3–5 days. While our human and rat sepsis studies involved systemic infection, SARS-CoV-2 infection of K18 mice initially was primarily localized to infection of the respiratory tree. Infection led to robust increases in the expression of pro-inflammatory cytokines. The loss of plasmalogen in the lung during SARS-CoV-2 infection likely is the result of oxidative stress. We did not observe a loss in DPPC in the lung of infected mice. The chemical makeup of plasmalogens compared to DPPC provides a contrast in susceptibility to oxidative stress. The plasmalogen vinyl ether bond is a target for oxidation while the saturated fatty acids of DPPC are very stable under oxidative stress. Similar to findings with severe COVID-19 patients (Schwarz et al., 2021; Snider et al., 2021) we detected decreases in plasma plasmalogens in infected K18 mice.

This is the first demonstration of the loss of plasmalogens at a molecular species level in human sepsis. Furthermore, we show pLPC loss in both human and rodent sepsis. It is possible that plasma pLPC is a critical lipid to maintain endothelial plasmalogen levels under oxidative stress associated with sepsis. The demonstration of plasmalogen loss during SARS-CoV-2 further highlights the nature of plasmalogen loss during oxidative stress associated with infectious disease. The role of plasmalogens as biomarkers of outcomes in sepsis and COVID-19 need to be explored as well as the potential protective role of plasmalogens during infectious disease.

## Data Availability

The original contributions presented in the study are included in the article/Supplementary Material, further inquiries can be directed to the corresponding author.
