# Supplementary material for: Plasmalogen Loss in Sepsis and SARS-CoV-2 Infection
Source: Front Cell Dev Biol. 2022 Jun 6;10:912880. doi: 10.3389/fcell.2022.912880 (PMC9242022; doi:10.3389/fcell.2022.912880)
Supplement: Supplementary file 1 [file Table1.DOCX]

**_­­­_**

**Table S1**

| **pPC and pLPC molecular species SRM** | **Precursor *m/z***  **[M+H]^+^** | **Product ion *m/z*** | **Polarity** | **CE** |
| --- | --- | --- | --- | --- |
| p16:0-18:1 | 744.6 | 184 | Positive | 30 |
| p16:0-18:2 | 742.6 | 184 | Positive | 30 |
| p16:0-20:4 | 766.6 | 184 | Positive | 30 |
| p16:0-22:6 | 790.6 | 184 | Positive | 30 |
| p18:0-18:2 | 770.6 | 184 | Positive | 30 |
| p18:0-20:4 | 794.6 | 184 | Positive | 30 |
| p18:0-22:6 | 818.6 | 184 | Positive | 30 |
| Diacyl PC 20:0 (Int Std) | 846.7 | 184 | Positive | 30 |
| p16:0 (pLPC) | 480.3 | 184 | Positive | 30 |
| p18:0 (pLPC) | 508.4 | 184 | Positive | 30 |
| Monoacyl LPC 17:0 (Int Std) | 510.4 | 184 | Positive | 30 |
| **pPE molecular species SRM** | **Precursor *m/z***  **[M-H]^-^** | **Product ion *m/z*** | **Polarity** | **CE** |
| p16:0-20:4 | 722.5 | 196 | Negative | 35 |
| p16:0-22:6 | 746.5 | 196 | Negative | 35 |
| p18:0-18:2 | 726.5 | 196 | Negative | 35 |
| p18:0-20:4 | 750.5 | 196 | Negative | 35 |
| p18:0-22:6 | 774.5 | 196 | Negative | 35 |
| Diacyl PE 14:0 (Int Std) | 634.4 | 196 | Negative | 35 |

**Table S1**: **Parent ion and product ion *m/z* used in selected reaction monitoring (SRM).** CE: collision energy, Int Std: internal standard, pPC: plasmenylcholine, pLPC: lysoplasmenylcholine, pPE: plasmenylethanolamine
